# Atypical Femur Fractures—An Analysis of 69 Patients from 15 Years

**DOI:** 10.3390/jcm14072404

**Published:** 2025-04-01

**Authors:** Cheuk Kin Kwan, Ning Tang, Man Ki Fong, Wing Hong Liu, Chi Yin Tso, Chaoran Liu, Pui Yan Wong, Ning Zhang, Wing Hoi Cheung, Ronald Man Yeung Wong

**Affiliations:** 1 Department of Orthopaedics and Traumatology, The Chinese University of Hong Kong, Hong Kong, China; 2 Department of Orthopaedics and Traumatology, Prince of Wales Hospital, Hospital Authority, Hong Kong, China

**Keywords:** atypical femur fracture, bisphosphonates, osteoporosis, rehabilitation

## Abstract

**Background/Objectives**: Bisphosphonates are effective in preventing osteoporotic fractures. However, the risk of atypical femur fractures (AFFs) increases with long-term bisphosphonate use. There are few existing publications on the analysis of clinical outcomes of atypical femur fracture cases in Chinese patients. Our objective was to review the clinical outcomes of AFF cases managed in a tertiary center in Hong Kong, China. **Methods**: Cases of AFF managed in the Prince of Wales Hospital from 2010 to 2024 were included. Data on demographics, type and duration of bisphosphonate use prior to AFF, fixation method, and mobility 1 year post-operation were retrospectively retrieved. One-way ANOVA was used to compare the duration of use prior to the development of AFF between different types of bisphosphonates. **Results**: Sixty-nine cases of AFF were included, with a mean age of 73.8 ± 9.7 years. A total of 95.6% of patients had a history of bisphosphonate use, with a mean duration of usage of 6.8 ± 5.6 years prior to the occurrence of AFF. The duration of bisphosphonate use prior to the development of AFF was comparable between alendronate, ibandronate, and a history of using more than one type of anti-resorptive agent. A non-union rate of 5.8% was observed in the current cohort, with 48.2% returning to pre-morbid mobility 1 year post-operation. **Conclusions**: AFF is more commonly seen in female patients with a history of bisphosphonate use. Considering the high success rate demonstrated in the current cohort, treating AFF with closed reduction followed by fixation with a long cephalomedullary device in dynamic locking together with immediate full-weight-bearing rehabilitation post-operation may be effective.

## 1. Introduction

Osteoporosis is a systemic skeletal disease involving bone tissue, leading to bone fragility and susceptibility to fractures [1,2], and it is common amongst the aging population. Fracture liaison services (FLSs) have been implemented worldwide as an effective method to reduce the risk of osteoporotic fractures [3] by actively recruiting and treating patients with osteoporosis. Their effectiveness in reducing fracture risk has been reported in previous studies [3,4,5].

Bisphosphonates have been widely used to treat osteoporosis since their development. In brief, bisphosphonates reduce bone resorption by inhibiting osteoclast activity [6]. Other anti-resorptive agents include denosumab, which is a monoclonal antibody that binds with the RANKL protein, preventing it from stimulating essential pathways by which osteoclast precursor cells mature into osteoclasts [7]. Rare adverse events from anti-resorptive use have been described in the literature, including atypical femoral fracture (AFF), which can occur as a result of anti-resorptive use. It typically presents as a transverse fracture of the subtrochanteric region, with minimal comminution occurring without a significant trauma history [8].

Previous studies suggested that the incidence of atypical femur fractures is low, ranging from 0.2 to 13 per 10,000 patient years, depending on duration of use [9]. However, most reported cases have been associated with oral alendronate, and whether a similar pattern is observed with other oral bisphosphonates or denosumab remains unclear [6]. It has also been reported in the literature that the risk of atypical femoral fracture drops significantly after discontinuation of bisphosphonates [9]. Therefore, “drug holidays” after every few years of treatment have been recommended for cases of long-term treatment [6].

AFFs are hypothesized to result from changes in the bone caused by decreased bone turnover [10] and microcrack formation due to impaired healing [11,12]. Reduced bone turnover is observed in anti-resorptive agent use, but it is also observed in patients with other conditions, such as diabetes and chronic kidney disease [13,14]. Previous research has suggested that Asian ethnicity is a risk factor for the development of AFF compared to Caucasian ethnicity [15]. However, a recent article also suggested that the incidence in the Korean population may be comparable to that reported for other ethnicities [16,17]. It was also reported that AFF is a challenging condition to manage, with an average time to union of 10.7 months [18], resulting in high complication rates reaching 15% [19], requiring revision surgery [20]. Currently, dedicated studies describing AFF in our locality and in the Chinese population are still scarce in the literature, and the clinical outcomes in this population may be different to those in the Western population. The primary objective of this study was to present the clinical outcomes of AFF cases managed in a tertiary unit in Hong Kong, China, and to compare the cases based on the duration, type, and practice of pharmacological agent used.

## 2. Materials and Methods

### 2.1. Patient Recruitment

This was a retrospective study approved by the Joint Chinese University of Hong Kong—New Territories East Cluster Clinical Research Ethics Committee (CRE Ref. No.: 2024.359, approval date: 20 August 2024). The study protocol was in compliance with ICH-GCP and the Declaration of Helsinki. Sixty-nine consecutive patients with atypical femur fractures (AFFs) who were admitted to the Prince of Wales Hospital in Hong Kong, China, from 2010 to 2024 were included. The inclusion criteria were (1) both male and female patients, (2) >= 18 years of age, and (3) diagnosis of atypical femur fracture based on the American Society for Bone and Mineral Research (ASBMR) guidelines. Closed reduction followed by fixation with a long cephalomedullary device is the usual practice when encountering cases of AFF. Full weight-bearing is allowed post-operatively [5,11]. Prophylactic nailing of the contralateral femur was discussed with patients when there were clinical signs or obvious stress lesions seen in radiographs. Cases of femur fracture that did not fulfill the ASBMR criteria for AFF were excluded.

### 2.2. Data Collection

Clinical notes of recruited cases were retrieved from the Clinical Management System (CMS) for demographics, Age-Adjusted Charlson Comorbidity Index (ACCI) [2,21], type and duration of osteoporosis medication used, documentation of “drug holidays”, treatment of choice for atypical femur fracture, whether prophylactic nailing of the contralateral femur was performed, and functional mobility premorbid and 1 year post-operation were extracted [22].

### 2.3. Statistical Analysis

Demographic variables were presented with means ± standard deviations (SDs) and frequencies (percentages). The Kolmogorov–Smirnov test was used for normality analyses. The functional scores between cases with and without prophylactic nailing of the contralateral femur were compared using independent-sample *t*-tests. The duration of use between different types of bisphosphonates prior to the development of AFF was compared using one-way ANOVA. A post hoc Scheffe’s test was performed if significant differences were obtained. Data were analyzed using IBM SPSS Statistics, version 27 (SPSS Inc., Chicago, IL, USA). Statistical significance was indicated with *p* < 0.05.

### 2.4. Outcome Measures

The primary outcomes of the current study were the clinical outcomes of 69 AFF cases in terms of mobility at 1 year post-operation, complication rates, and mortality. Secondary outcomes included the type and duration of anti-resorptive agent used and the practice of drug holidays.

## 3. Results

### 3.1. Patient Demographics

Clinical notes of 69 patients with a diagnosis of atypical femur fracture from 2010 to 2024 were reviewed. The patients had a mean age of 73.8 ± 9.7 years old, a BMI of 23.8 ± 3.9 kg/m^2^, and a female ratio of 94.2%. A total of 21.7% had a history of diabetes mellitus, 7.2% had a documented history of smoking, 4.3% had heart failure, 4.3% had chronic kidney disease, and 8.7% had a history of malignancy. The mean Age-Adjusted Charlson Co-morbidity Index was 3.4 ± 1.2. A total of 53 out of 69 (76.8%) participants had a history of anti-resorptive agent use for primary osteoporosis, whilst 7 out of 69 (10.1%) initiated anti-resorptive treatment after fragility fracture. Six cases had a history of anti-resorptive agent use without clear documentation of the indication, and three cases had clear documentation that the participant had no previous use of anti-resorptive agents. The details of the recruited patients are shown in Table 1.

### 3.2. Fixation Method and Non-Union Rates

Amongst the 69 recruited cases, 51 (75.3%) were treated with a long cephalomedullary device (300 mm was the shortest one used). Sixteen (23.2%) were treated with a short cephalomedullary device (200 mm was the longest one used), and one (1.4%) was treated with a plate with screws.

Four out of sixty-nine cases (5.8%) resulted in non-union, requiring revision. Amongst these four cases, one case underwent fixation of AFF with a 360 mm long cephalomedullary device in static locking. The other three had initial fixation of AFF with other units: one case was initially treated with a 170 mm cephalomedullary device, one case was treated with a 200 mm cephalomedullary device, and one case was treated with plating of the proximal femur. All four cases were revised with 300–360 mm long cephalomedullary devices in dynamic locking. All cases achieved bony union after revision surgery. The non-union rates with different devices are summarized in Table 2.

### 3.3. Location of AFFs and Non-Union Rates

Amongst the 69 recruited cases of AFF, 22 were located in the diaphyseal region, while 47 were located in the sub-trochanteric region. Out of the four cases of non-union in this cohort, one (4.5%) case had an AFF in the diaphyseal region, while three (6.4%) cases had AFFs in the sub-trochanteric region. The non-union rates with AFFs in different locations are summarized in Table 3. There was no statistical significance in the difference between non-union rates of subtrochanteric and diaphyseal AFFs (*p* > 0.05).

### 3.4. Mobility Status 1 Year Post-Operation

**Figure 1:**
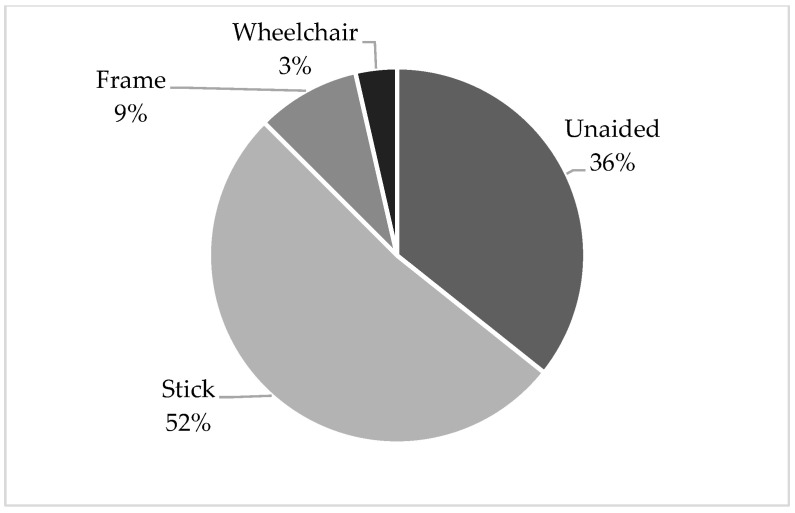
Mobility achieved at 1 year post-operation.

Amongst the 69 patients, 56 (81.8%) had information on mobility status at baseline and at 1 year post-operation documented. Twenty-seven (48.2%) cases were able to return to premorbid mobility levels at 1 year post-operation. Twenty (35.7%) cases walked unaided, twenty-nine (51.8%) cases walked with a stick, five (8.9%) cases walked with a frame, and two (3.6%) cases were wheelchair-bound (Figure 1).

### 3.5. Prophylactic Nailing

Prophylactic nailing of the contralateral femur was performed in 78.2% (54 out of 69) of recruited cases of AFF. At 1 year post-operation, there was no documented occurrence of AFF of the contralateral femur among cases without prophylactic nailing performed. There were also no significant differences in the functional mobility status of AFF cases with and without prophylactic nailing of the contralateral femur performed (*p* > 0.05).

### 3.6. Pharmacological Agents Used for Osteoporosis

A total of 66 out of 69 (95.6%) patients had a documented history of using anti-resorptive agents, with a mean duration of 6.8 ± 5.6 years, prior to the development of AFF. Alendronate was the most common pharmacological agent used, documented in 42.0% of included cases. Ibandronate was used in 8.6%. Denosumab was used in 4.3% of cases. A total of 14.4% of AFF cases had a history of using more than one type of medication for osteoporosis prior to the development of AFF, and 26.1% cases had no clear documentation on the type of anti-resorptive agent used. The details of anti-resorptive agents used in the included population are summarized in Table 4.

**Figure 2:**
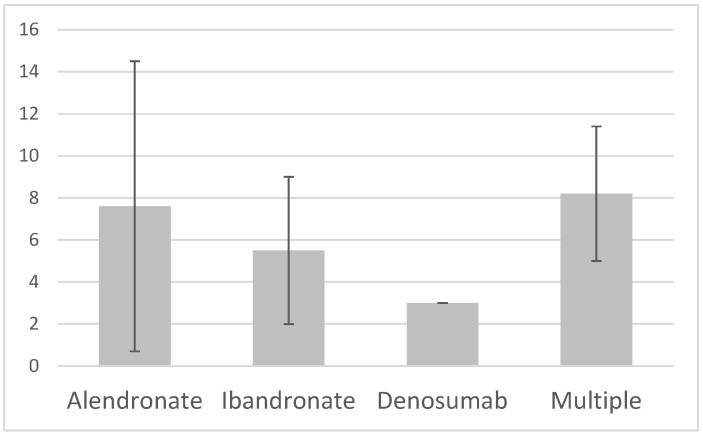
Years of anti-resorptive agent use prior to the development of atypical femur fractures (AFFs).

The documentation of the duration of drug use was inconsistent, with retrievable data in 53 out of 69 cases (76.8%). Missing data included the type, duration, and indication of anti-resorptive agent used. The cause of incomplete documentation was the use of over-the-counter anti-resorptive agents or the participants receiving medication from a private practice. AFF patients with sole alendronate use developed the condition after 7.6 ± 6.9 years. Patients with sole Ibandronate use developed AFF after 5.5 ± 3.5 years of treatment. Three AFF cases had a history of denosumab use, with a mean time of 3 ± 0 years use prior to development of AFF. There were no significant differences in the duration of use prior to the development of AFF observed between different types of osteoporosis medications (Figure 2).

### 3.7. Drug Holidays

Amongst the 53 participants with available data on drug holidays, 37 cases had a history of anti-resorptive use of more than 4 years. Only 3 out of 37 (8.1%) cases practiced a drug holiday of 1–2 years after every 3 years of consecutive use of anti-resorptive agents. In these cases of AFF, the average time of consecutive anti-resorptive agent use was 8.9 ± 5.7 years.

## 4. Discussion

### 4.1. Comparable Non-Union Rates in the Literature

**Figure 3:**
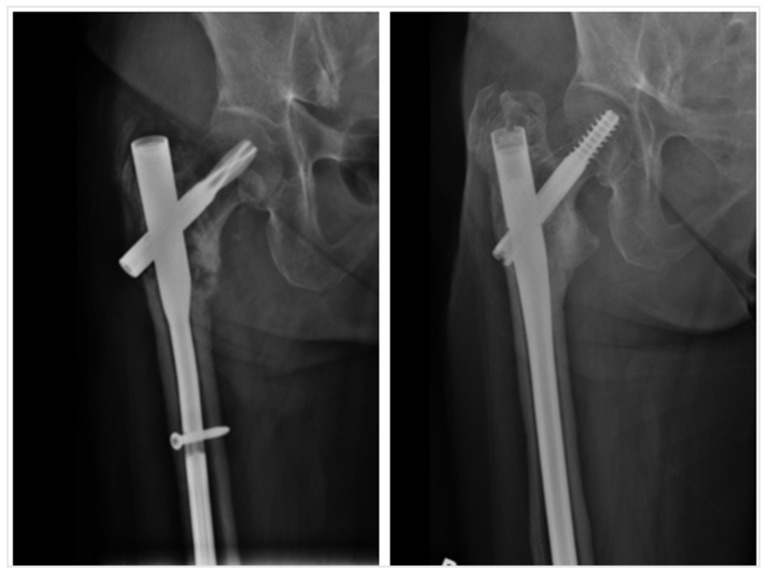
Case of AFF with non-union after fixation with short cephalomedullary device revised to long cephalomedullary device and achievement of bony union.

A previous multicenter study reported a cohort of 46 AFF cases treated with intramedullary nailing, resulting in a 4.3% non-union rate of requiring revision of intramedullary nailing [23]. In this study, 4 out of 69 (5.8%) cases of AFF required revision surgery, all of which achieved boney union after. This suggests that the non-union rate in the current study is comparable to that reported in the literature. Previous studies recommended that AFF should be treated by closed reduction followed by fixation with a long cephalomedullary device, providing adequate stability to allow immediate weight-bearing [24]. The findings of the current study support this recommendation, as non-union rates were shown to be higher in cases treated with a short cephalomedullary device (12.5%) or a plate with screws (100%). Amongst our four included cases of non-union, two cases initially attempted fixation of AFF with a short cephalomedullary device, and one case attempted fixation with plates and screws. Shorter cephalomedullary devices may not provide adequate stability, whilst extramedullary fixation methods like plates and screws may be sub-optimal as they rely on intramembranous fracture healing, which may be inhibited by anti-resorptive medications. As for the one case of failed fixation with a long cephalomedullary device, the distal locking was performed in the static position, which may have contributed to its failure (Figure 3). Static locking of cephalomedullary devices for fixation of AFF may lead to higher rates of failure due to the limited distance available for controlled subsidence. A previous study described static locking as one of the risk factors of reoperation [25].

Another explanation for the high rate of bony union in the current cohort (94.2%) could be our routine practice of dynamic locking together with immediate full weight-bearing post-operation. Comparisons of static locking and dynamic locking in the treatment of AFF are not widely reported in the existing literature. However, the potential benefits of dynamic locking were highlighted in a recent cohort study of 236 cases of AFF showing faster times for the achievement of union and lower non-union and failure rates [25].

Dynamic locking is typically avoided in cases of comminuted or long oblique cases where a lack of fracture end opposition may lead to significant limb shortening [26,27,28] and delayed time to union [29,30,31], but the fracture patterns in AFFs are typically transverse or short oblique with minimal or no comminution. When adopted in suitable cases, it is believed that dynamic locking may stimulate an osteogenic response due to increased load across the fracture site [32]. No existing large-scale randomized trials compared the use of different locking options in the treatment of AFF. However, clinical trials have been performed on cases of femur fractures, showing mixed results. One study suggested that dynamic locking achieved a shorter time to union by 4 weeks [33], while another study suggested that static locking achieved a shorter time to union by 3 weeks [34]. The results from the existing literature may not be directly comparable due to differences in the recruited populations and differences in fracture patterns and rehabilitation protocols.

The results from the current study showed only one failed fixation of AFF using a long cephalomedullary device, which was fixed in static locking. There is a possibility that dynamic locking may provide superior outcomes, but further investigation is required to support this hypothesis.

### 4.2. Non-Union Rates Between Diaphyseal and Subtrochanteric AFFs

Previous studies suggested the classification of AFFs into diaphyseal and subtrochanteric types [8]. The clinical significance of this classification has also been highlighted in a recent systematic review showing a statistically greater non-union rate of subtrochanteric AFF at 15% versus a non-union rate of 4% in diaphyseal AFF [19]. A similar trend was observed in the cohort of the current study. Despite not reaching statistical significance, the non-union rate of subtrochanteric AFFs was higher than that of diaphyseal AFFs (6.4 vs. 4.5%). It is also noteworthy that the union rate of subtrochanteric AFFs in this study (6.4%) was lower than the non-union rate of 15% reported in the literature. Prospective studies with a larger sample size are required to support the hypothesis, but there is a possibility that the standardized AFF treatment with a long cephalomedullary device and dynamic locking followed by immediate full-weight-bearing walking may be a reasonable method to manage the challenging condition.

### 4.3. Mobility at 1 Year Post-Operation

In the current study, 56 (81.8%) recruited cases documented information on mobility status at baseline and at 1 year post-operation. Twenty-seven (48.2%) cases were able to return to premorbid mobility levels at 1 year post-operation. A previous multicenter study on 75 cases of AFF suggested that 80.4% of patients were able to return to pre-fracture mobility [23,35]. The discrepancy could be addressed by the relatively short follow-up time in the current study. Documentation of pre-fracture and post-fracture mobility could also be documented in a more standardized manner in future studies.

### 4.4. Significance of Prophylactic Nailing to the Contralateral Femur

Existing multicenter cohorts suggested the effectiveness of intramedullary nailing in treating atypical femur fractures [23,36]. Extramedullary fixation with plates and screws was also described in the literature but was suggested to be un-favourable as intramembranous fracture healing is dependent on osteoclast activity, which is inhibited in anti-resorptive therapy [5]. The management of incomplete fractures and whether to prophylactically perform intramedullary nailing of the contralateral femur remain controversial [24]. A scoring system has been described in previous literature to guide the decision on whether prophylactic nailing should be performed [37].

**Figure 4:**
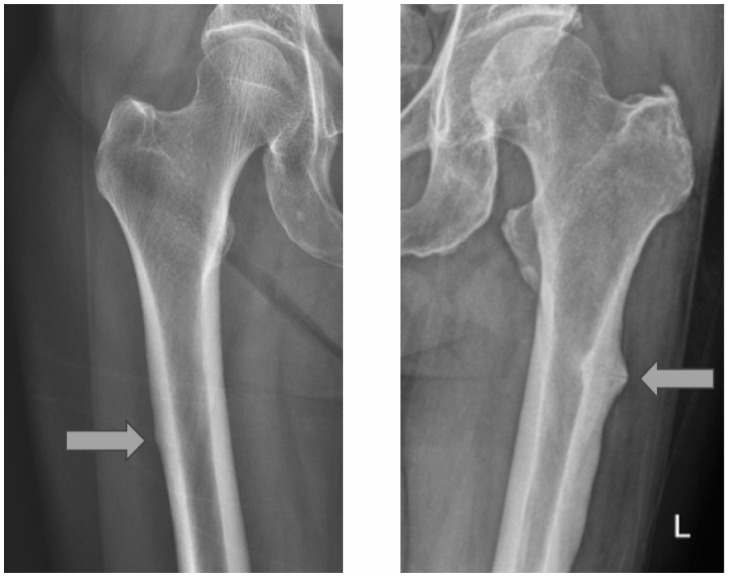
Example radiographs of cases where prophylactic nailing of the contralateral femur was (**right**) and was not performed (**left**). Grey arrows show the most prominent stress lesions on the radiographs.

Prophylactic nailing of the contralateral femur was offered to all patients presenting with AFF, as there were clinical signs or radiological stress lesions. In this study, 22% of the cases presenting with AFF did not receive prophylactic nailing of the contralateral femur after discussion. The decision usually involves a balance of risks/benefits, patient preference, and discussion with the surgeon when there is a lack of significant stress lesioning suggested by radiography of the contralateral femur. An example of a significant stress lesion strongly suggestive for prophylactic nailing and a minimal stress lesion for which the patient eventually did not receive prophylactic nailing are shown in Figure 4.

When comparing cases with and without prophylactic nailing of the contralateral femur, there were no occurrences of AFF in the contralateral femur and no significant differences in the functional mobility status at 1 year post-operation. These results raise awareness of the fact that prophylactic nailing may not necessarily be beneficial in cases of AFF when there is a lack of significant stress lesions shown on the radiograph of the contralateral femur.

### 4.5. Time Leading to AFF in Different Anti-Resorptive Agents

The existing literature suggested that the majority of reported cases of AFF were associated with alendronate use, but the phenomenon was also observed in trials involving other anti-resorptive agents [6]. The etiology of AFF is yet to be fully understood. Anti-resorptive agents aim to preserve bone mineral density through suppression of bone resorption. However, resorption is part of the natural bone turnover mechanism, and it was hypothesized that its suppression may lead to the accumulation of microcracks and AFF [38,39]. Previous investigations also showed depressed levels of bone turnover markers when comparing AFFs with typical femur fractures [10]. Other studies attempted to compare the microarchitecture of bones in patients with and without atypical femur fractures, showing no significant differences [40]. Results from this study suggested that AFF cases are also observed in cases of anti-resorptive agent use other than alendronate. From the current data, there were no significant differences in the time leading to AFF between using alendronate only, ibandronate only, denosumab only, or a history of more than one type of anti-resorptive agent used prior to the development of AFF. However, the current study only included a relatively small sample of AFF patients. Population-based studies with a larger sample size may be required to further support this hypothesis.

### 4.6. The Importance of Stewardship in the Use of Anti-Resorptive Agents

The results from this study suggest that drug holidays were not practiced according to recommendations in the majority (92%) of cases presenting with AFF. Among these cases, the mean duration of consecutive anti-resorptive agent use was 8.9 ± 5.7 years. The importance of drug holidays has been advocated in the previous literature [41]. Existing studies demonstrated that bone turnover increases gradually over 1–2 years, leading to a drop in bone mineral density in the hips [42]. However, the bone mineral density at 2 years post-termination of anti-resorptive treatment was still higher than pre-treatment levels, suggesting lingering effects by the end of a drug holiday of 2 years [42]. Another observational cohort study assessing the fracture risks of drug holidays reported an increased risk of hip, humerus, and vertebral fractures in patients with a drug holiday of >2 years compared with patients who continued to receive anti-resorptive agents [43]. Despite evidence on the importance of drug holidays, there is currently no consensus on when and how long drug holidays should be practiced. Large, prospective, long-term observational studies may be necessary to clarify the safety of and best practice for drug holidays.

As the risk of typical fragility fracture remains high in patients with osteoporosis, five principles were described to manage patients who suffered from AFF while on bisphosphonates or denosumab [44]. The first principle suggested the termination of the anti-resorptive medication. However, it was also mentioned that extra caution is required for patients on denosumab in consideration of the risk of rebound vertebral fractures [44]. The second principle is maximizing non-pharmacological means to reduce fall and fracture risks. The third principle is to work-up and treat potential secondary causes of osteoporosis. The fourth principle is to review the drug list of the patient, eliminating candidates that may increase fracture risks. Finally, the fifth principle suggested that some patients may benefit from anabolic agents. The use of anabolic agents like teriparatide was also recommended by other authors [45]. However, it was also mentioned that there is currently no solid evidence on the indication of anabolic agents [45].

### 4.7. Limitations

A limitation of the current study lies in the relatively small sample size of 69 cases of AFF and the retrospective study design. Documentation of the use of medication for osteoporosis is sometimes inconsistent, and the variety of anti-resorptive agents discussed in the current study is limited to alendronate, ibandronate, and several cases of denosumab. Inconsistent documentation of anti-resorptive agent use also limited the possibility of performing a meaningful analysis of the spontaneous or non-bisphosphonate groups of AFF, as accurate identification of these groups could not be completed with the current data.

The recruited cohort of the current study only included Chinese patients. Therefore, direct comparisons between different ethnicities could not be made. The retrospective design of the current study also limited the width of the included data. Frailty measures, muscle mass, and physical function are important parameters to be considered in the geriatric population. Future prospective multicenter studies on the topic with prospective designs may be required given the rare incidence of AFF due to anti-resorptive agent use.

## 5. Conclusions

AFF is more commonly seen in female patients with a history of bisphosphonate use. Considering the high success rate demonstrated in the current cohort, treating AFF with closed reduction followed by fixation with a long cephalomedullary device in dynamic locking together with immediate full-weight-bearing rehabilitation post-operation may be effective.

## Figures and Tables

**Table 1:** Age distribution of cases presenting with atypical femoral fracture.

Characteristics	Total Cases (*n* = 69)
Age (years) ^a^	73.8 ± 9.7
Female	65 (94.2%)
BMI	23.8 ± 3.9 kg/m^2^
Current smoker	5 (7.2%)
Diabetes mellitus	15 (21.7%)
Heart failure	3 (4.3%)
Chronic kidney disease	3 (4.3%)
Malignancy ^b^	6 (8.7%)
ACCI ^c^	3.4 ± 1.2
History of anti-resorptive agent use	66 (95.6%)
Indication for anti-resorptive agent	
Primary osteoporosis	53 (76.8%)
Fragility fracture	7 (10.1%)

^a^ Values are expressed as means ± standard deviations or n (%). ^b^ Documentation of any previous/active malignancy. ^c^ Age-Adjusted Charlson Comorbidity Index.

**Table 2:** Non-union rates with different devices for fixation.

	Union	Non-Union	Total
Long cephalomedullary device	51 (98.1%)	1 (1.9%)	52
Short cephalomedullary device	14 (87.5%)	2 (12.5%)	16
Plate with screws	0 (0%)	1 (100%)	1
Total	65 (94.2%)	4 (5.8%)	69

**Table 3:** Non-union rates of AFFs in different locations.

	Union	Non-Union	Total
Subtrochanteric	44 (93.6%)	3 (6.4%)	47
Diaphyseal	21 (95.5%)	1 (4.5%)	22
Total	65 (94.2%)	4 (5.8%)	69

**Table 4:** Type of anti-resorptive agent used in recruited population.

	Total Cases (*n* = 69)
Cases with documented use of anti-resorptive drugs	66 (95.6%)
Type of anti-resorptive agent	
Alendronate only	29 (42.0%)
Ibandronate only	6 (8.6%)
Denosumab only	3 (4.3%)
History of using more than one drug	10 (14.4%)
Unknown	18 (26.1%)

Values are expressed as *n* (%).

## Data Availability

Data are contained within the article. Individual data are unavailable due to privacy or ethical restrictions.

## Abbreviations

The following abbreviations are used in this manuscript:

ACCI	Age-Adjusted Charlson Comorbidity Index
AFF	Atypical femur fracture
CMS	Clinical Management System
FLS	Fracture liaison service
